# Anti-Inflammatory MicroRNAs and Their Potential for Inflammatory Diseases Treatment

**DOI:** 10.3389/fimmu.2018.01377

**Published:** 2018-06-25

**Authors:** Alireza Tahamtan, Majid Teymoori-Rad, Britt Nakstad, Vahid Salimi

**Affiliations:** ^1^Infectious Diseases Research Centre, Golestan University of Medical Sciences, Gorgan, Iran; ^2^Department of Microbiology, School of Medicine, Golestan University of Medical Sciences, Gorgan, Iran; ^3^Department of Virology, School of Public Health, Tehran University of Medical Sciences, Tehran, Iran; ^4^Department of Pediatric and Adolescent Medicine, Akershus University Hospital, Lørenskog, Norway; ^5^Institute of Clinical Medicine, University of Oslo, Oslo, Norway

**Keywords:** inflammation, immune regulation, microRNA, anti-inflammatory microRNA, inflammatory diseases

## Abstract

Inflammation is a complicated biological and pathophysiological cascade of responses to infections and injuries, and inflammatory mechanisms are closely related to many diseases. The magnitude, the complicated network of pro- and anti-inflammatory factors, and the direction of the inflammatory response can impact on the development and progression of various disorders. The currently available treatment strategies often target the symptoms and not the causes of inflammatory disease and may often be ineffective. Since the onset and termination of inflammation are crucial to prevent tissue damage, a range of mechanisms has evolved in nature to regulate the process including negative and positive feedback loops. In this regard, microRNAs (miRNAs) have emerged as key gene regulators to control inflammation, and it is speculated that they are fine-tune signaling regulators to allow for proper resolution and prevent uncontrolled progress of inflammatory reactions. In this review, we discuss recent findings related to significant roles of miRNAs in immune regulation, especially the potential utility of these molecules as novel anti-inflammatory agents to treat inflammatory diseases. Furthermore, we discuss the possibilities of using miRNAs as drugs in the form of miRNA mimics or miRNA antagonists.

## Introduction

**Figure 1:**
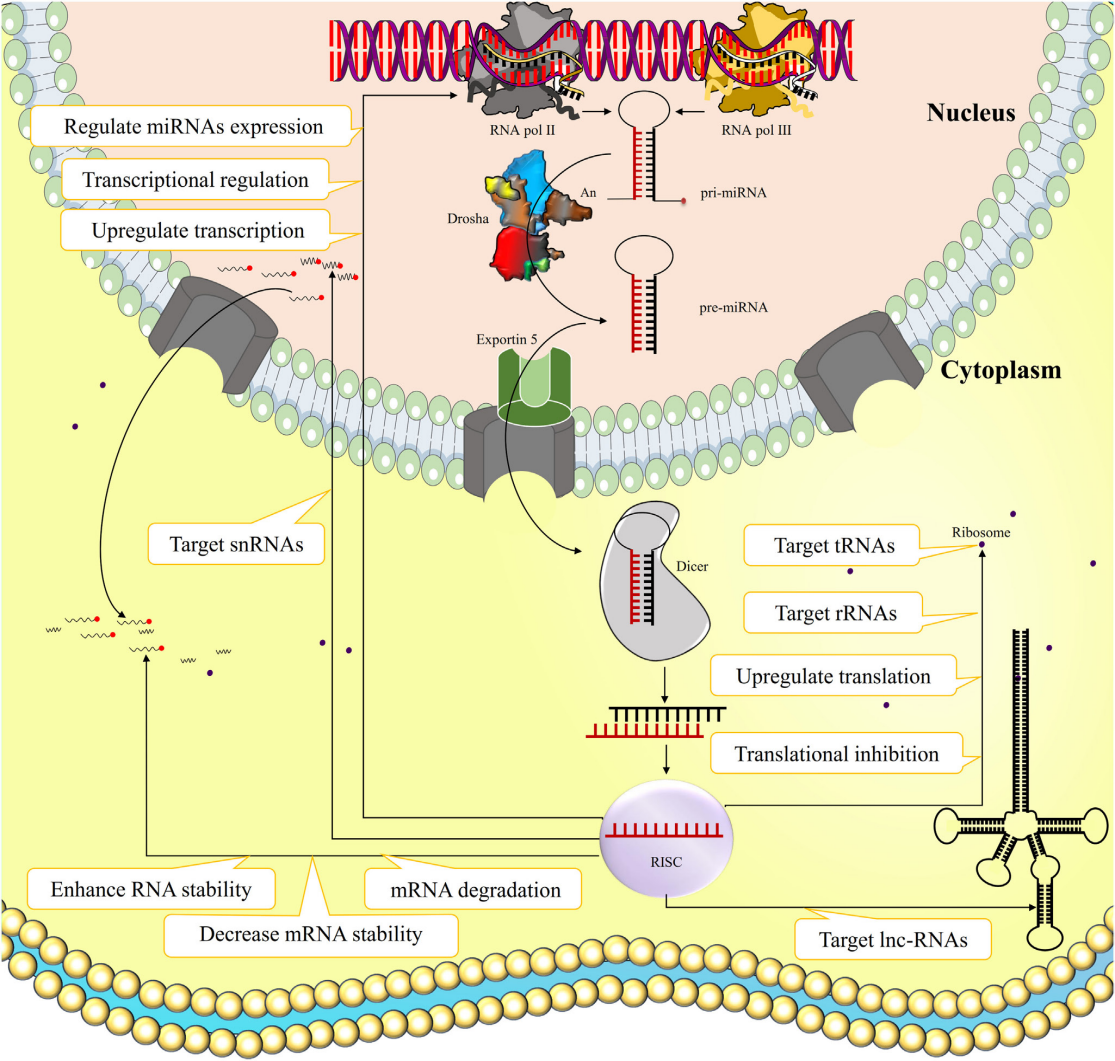
The biogenesis and function of microRNAs (miRNAs). miRNA is a short non-coding ribonucleic acid originated inter- or intragenicaly by RNA pol III and II, respectively. After initial processing by RNase Drosha in the nucleus, the pre-miRNA is transported to the cytoplasm where the miRNA hairpin is cleaved by endoribonuclease Dicer, forming an miRNA duplex. One of the miRNA strands loads into the RNA inducing silencing complex (RISC) that regulates mRNA transcription and protein translation through various ways. The miRNA binding to its target usually results in mRNA degradation, decreased mRNA stability, or translational inhibition, and hence they can influence transcriptional regulation of target genes. Alternatively miRNAs occasionally can enhance RNA stability and even upregulate transcription and translation of their certain targets. Some evidence also indicated that miRNAs can target long-non-coding RNAs (lncRNAs), ribosomal RNAs (rRNAs), transfer RNAs (tRNAs), and small nuclear RNAs (snRNAs), and regulate expression of other miRNAs.

MicroRNAs (miRNAs) are short non-coding RNA molecules usually composed of 18–25 nucleotides that originates inter- or intragenicaly by the action of RNA pol III and II, respectively (1–3). After initial processing by RNase Drosha in the nucleus, the pre-miRNA is transported to the cytoplasm, where the miRNA hairpin is cleaved by the endoribonuclease Dicer, forming an miRNA duplex. One of the miRNA strands is loaded into the RNA inducing silencing complex (RISC), which regulates mRNA transcription and protein translation through various ways (Figure 1). The miRNA binding to its target usually results in mRNA degradation or translational inhibition, and hence they can influence transcriptional regulation of target genes (4). Alternatively, miRNAs occasionally can enhance RNA stability and even upregulate transcription and translation of their specific targets (5–7). There are also evidence indicating that miRNAs can target long-non-coding RNAs (lncRNAs), ribosomal RNAs, transfer RNAs, and small nuclear RNAs, and regulate expression of other miRNAs (8, 9). However, the functional consequences of such actions remain unknown.

The miRNAs are expressed in a wide variety of organs and cells, and regulate both pro- and anti-inflammatory actions. The latter is the focus of this review. It has been estimated that there are nearly 5,000–10,000 miRNAs in mammals, together forming an miRNA network, which controls the expression of over two-thirds all protein-coding genes (www.mirBase.com). This system acts as a complex regulatory network, where a single miRNA may be involved in regulating many mRNA targets, and one mRNA can also be targeted by more than one miRNA (10–12). The miRNA network plays a critical role in regulating gene expression in health and disease, is crucial for normal mammalian development, and regulates biological processes such as the cell cycle, proliferation, and apoptosis (13). miRNAs are essential regulators of hematopoiesis, immune cell development, immune responses, inflammation, and autoimmunity, providing a new therapeutic window (14). Dysregulation of miRNAs expression has been linked to a wide spectrum of human diseases such as developmental abnormalities, cancer, and autoimmune diseases (15). Here, we review recent findings on the role of miRNAs as anti-inflammatory agents, and their potential utility as novel therapeutics for the treatment of inflammatory diseases.

## miRNAs and Immune Regulation

**Figure 2:**
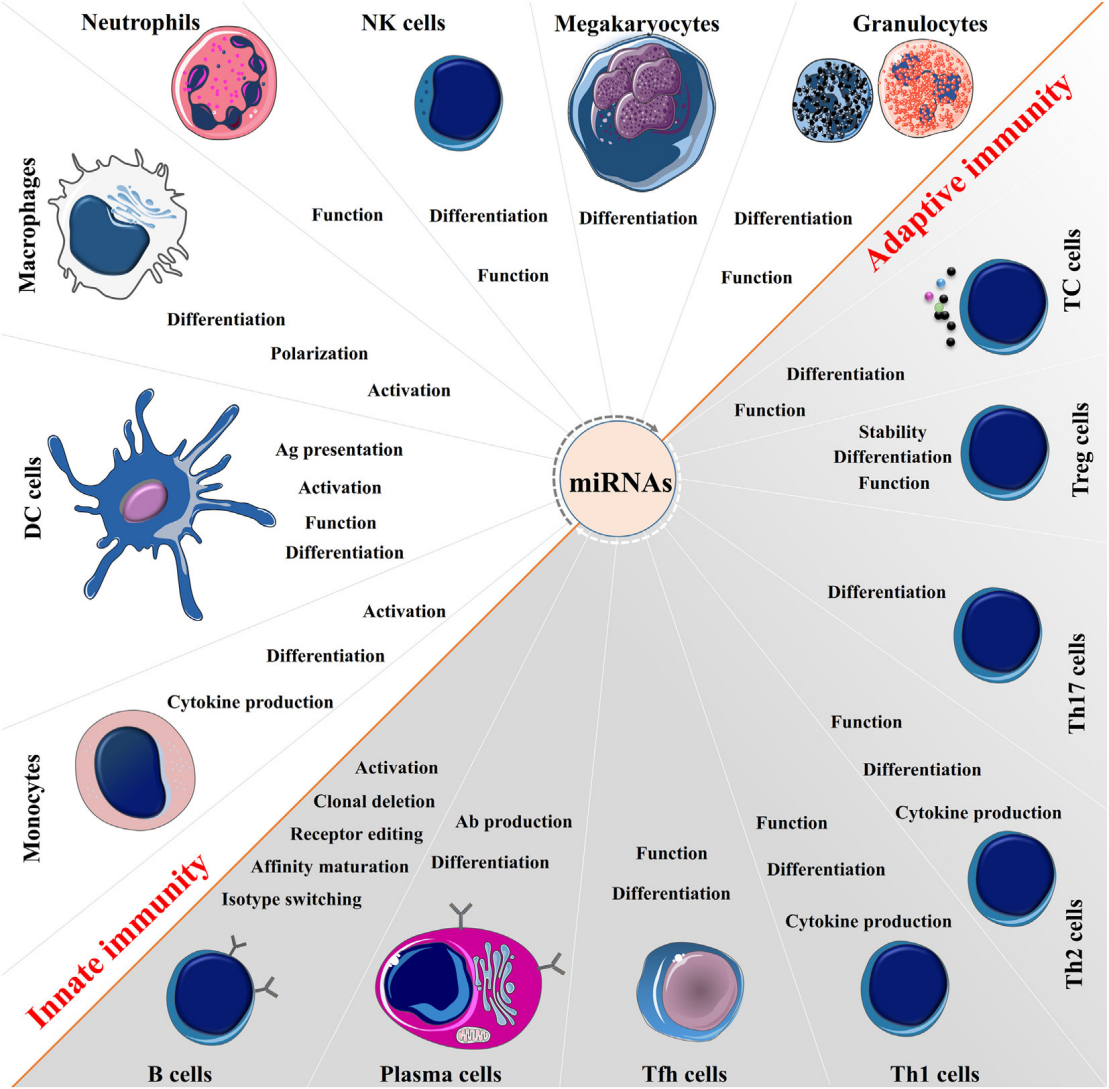
The broad function of microRNAs (miRNAs) in immune regulation. miRNAs are expressed in immune cells and function as “fine-tuners” for innate and adaptive immune responses. They establish an integrated part of the regulatory networks in innate immunity and regulate functions of immune cells such as monocytes, dendritic cells (DCs), macrophages, neutrophils, natural killer (NK) cells, megakaryocytes, and granulocytes. In adaptive immunity, they are implicated in every biological process including pathways involved in the T and B cells development, differentiation, central and peripheral tolerance, as well as their function.

Over the past decade, studies demonstrate that miRNAs function as “fine-tuners” of the immune system, playing a central role in the development and homeostasis of immune cells, which is important for the normal function of the immune responses (Figure 2). Importantly, some miRNAs such as miR-146 and miR-155 impact on activation of host defense pathways, which are linked to the control of immunity and the inflammatory sequelae (16). Previous studies elegantly demonstrated that a single miRNA can have a pivotal role in the development of both innate and adaptive immunity, and under some conditions might act as a negative feedback pathway, that modulates and impacts on immune dysfunction and disease (17). The mechanistic studies have suggested that this critical function is dependent upon interactions between miRNAs and transcription factors and targeting signaling proteins as well as regulators of cell death (18). Alternatively, the immune system can regulate biogenesis of miRNAs at multiple levels from transcription and microprocessing to loading into the RISC and localization of their action (19). Besides the essential role of miRNAs in directing the immune system, these molecules are also capable of acting as direct intracellular agent to combat against pathogens (20).

MicroRNAs are an integrated part of the regulatory networks in the innate immune response, acting as the first line of immunity. Activation of innate defense pathways such as toll-like receptor (TLR) signaling results in alterations in the expression of miRNAs that can regulate inflammatory gene expression (21). The dysregulated miRNAs can modulate translation of transcripts resulting in a decrease in the levels of immunomodulating factors that can inhibit or initiate the inflammatory response, thus acting as “on-off” brakes to regulate inflammation (22). Of particular interest is the central role that miR-146 plays in the control of TLRs and cytokine signaling through a negative feedback regulation loop (23). miRNAs can directly modulate the levels of molecules involved in the pattern-recognition receptors (PRR)-induced signaling, giving negative feedback in the PRR pathway (24). miRNAs also participate in the modulation of epithelial cell function (25), macrophages and dendritic cells (DCs) maturation (26, 27), granulocytes and monocytes proliferation (28), and natural killer (NK) cell function (29). Furthermore, miRNAs can regulate the expression of several cytokines/chemokines involved in the innate immune response (30).

MicroRNAs are key regulators of the development and generation of different T helper lineages and CD4^+^ T cell function (31). They also play a central role in the development, proliferation, survival, migration, differentiation, and effector functions of CD8^+^ T cells and regulatory T (Treg) cells (32, 33). In B cells, miRNAs appear to have a key role in the early and effector differentiation including isotype switching and affinity maturation as well as mature and memory cell responses (34, 35). Interestingly and related to autoimmune disease development, miRNAs are involved in receptor editing and clonal deletion to maintain T and B cells tolerance against self-antigens, thus their aberrant expression correlates with the onset and prognosis of many autoimmune conditions (15). As an example, dicer-deficient B cells produce high titers of autoreactive antibodies, which correlate with the presence of autoimmune features in animal models (36). miRNAs are also implicated in cytokine production by lymphocytes and antigen presentation by DCs (37). Moreover, miRNAs have the ability to regulate epigenetic condition in lymphocytes such as methylation, and amplify the strength and sensitivity of T- and B-cell receptor signaling (38).

The above facts indicate that the miRNA system has emerged as a critical regulatory network in several biological processes and involves both the innate and adaptive immune responses. This is illustrated in Figure 2, but it is beyond the scope of this review to discuss each miRNA and its relation to each immune cell in detail [for detail information please read the following articles (17, 39, 40)]. Below we focus on miRNAs that impact on inflammation through their anti-inflammatory properties.

## miRNAs and Inflammation

Inflammation is a complex biological and pathophysiological response induced by infection and/or tissue damage and involves a network of pro- and anti-inflammatory mediating molecules and effects (41, 42). The inflammatory response to a wide range of stimuli is a “double edged sword.” In its absence homeostasis cannot be resumed. On the other hand, inflammation may cause tissue damage, reversible or permanent, and induces disease processes (43, 44). Inflammation involves a harmonized, consecutive, and often self-limiting sequence of events controlled by positive and negative regulatory networks (41). Thus, the molecular networks that regulate the initiation, spread, and resolution of inflammation must be appropriately tuned for optimization of the innate immune response (40). Besides protein regulatory factors, miRNAs have emerged as key regulators of inflammation, and it is likely that they modulate signaling of onset and termination of inflammation. Depending upon the target mRNAs, miRNAs may either promote or suppress inflammation (40, 45). Therefore, the immune system utilizes multiple miRNAs to properly regulate its functional capacity thus establishing a fine balance between activation and inhibition (45). The interaction between miRNA function and inflammatory response is highlighted because this interaction can contribute to a better understanding on how depletion or downregulated immune homeostasis can be associated with autoimmunity conditions (46, 47).

**Figure 3:**
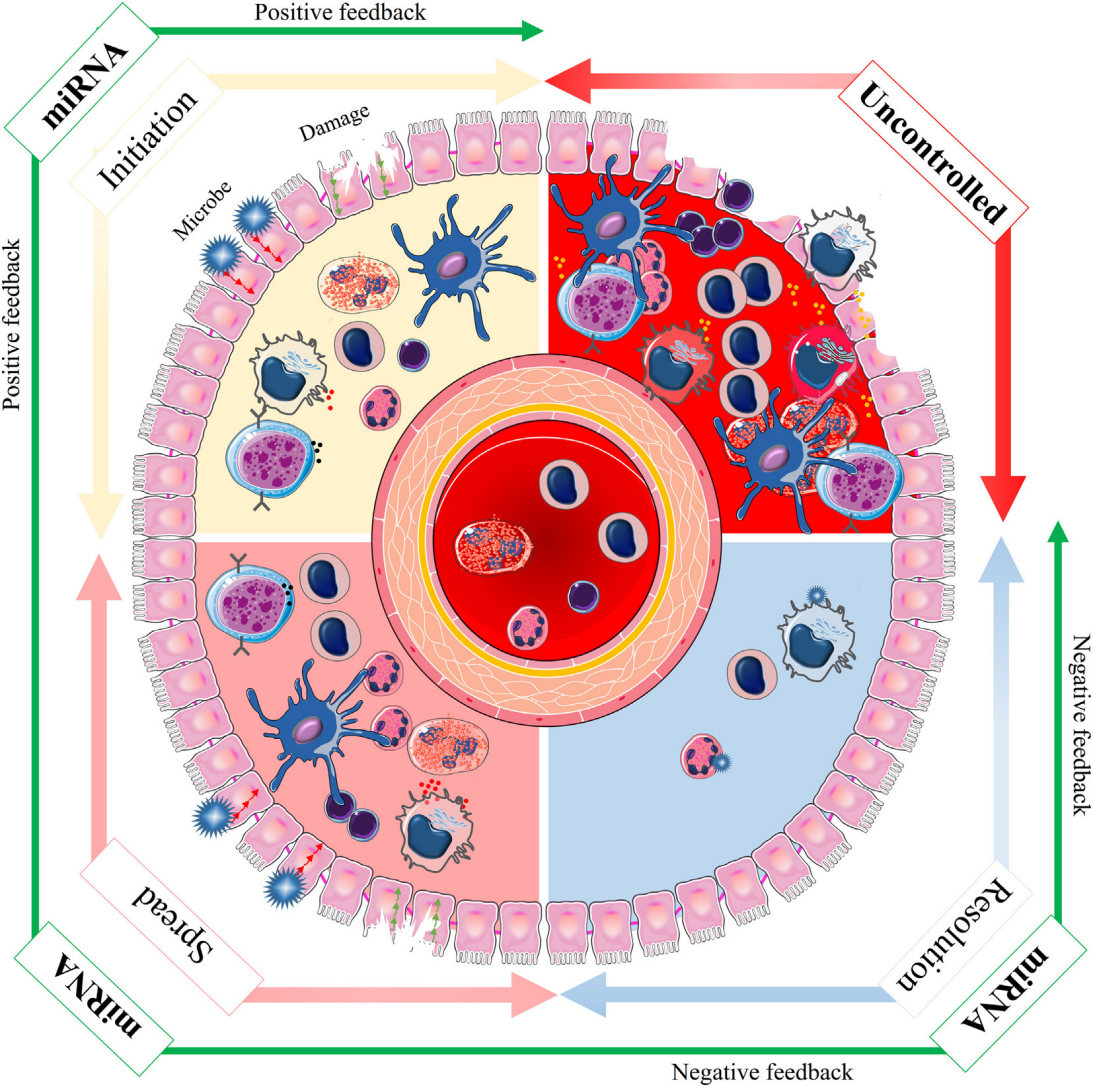
The spectrum of microRNA (miRNA) effects during inflammation. Inflammation is a complex biological and pathophysiological response in vascular tissues to noxious stimuli, such as infection and tissue damage. The initiation, spread, and resolution steps of inflammation are subject to both positive and negative regulatory events *via* miRNAs to achieve an optimal immune response (green arrow). The positive feedback is activated to initiate a cascade of molecular events that leads to combat invading microbial pathogens and successful repair of tissue damage. The negative feedback is only activated during severe inflammation and may be vital in preventing potentially dangerous and excessive inflammation. Lack of appropriate initiation or spread impedes the innate immune response, and lack of correct resolution can lead to uncontrolled condition and disease (red arrow). Thus, the molecular networks based on miRNAs that regulate the initiation, spread, and resolution of inflammation must be appropriately tuned for optimization of the innate immune response.

**Figure 4:**
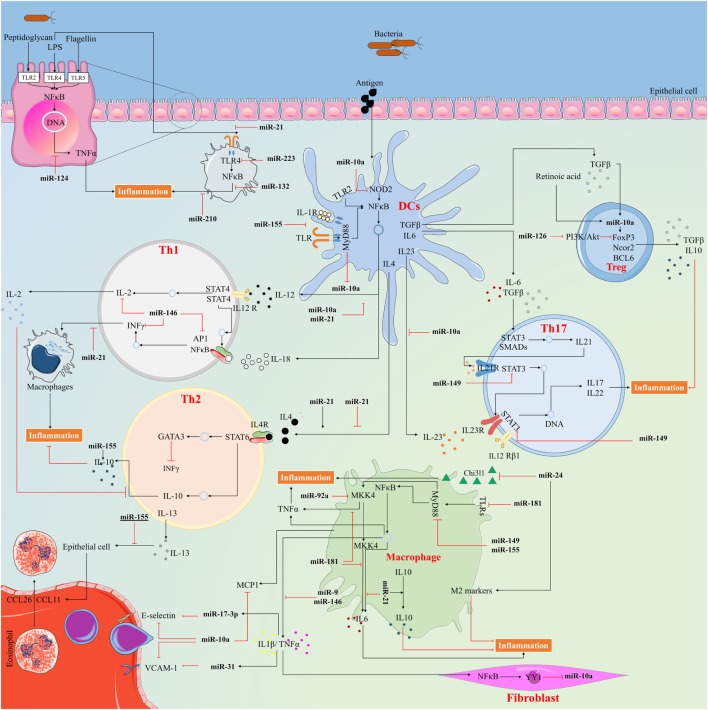
The way anti-inflammatory microRNAs (miRNAs) exert their action to control inflammatory response. miRNAs serve in important negative feedback loops in inflammation processes and inflammatory diseases. By targeting signal transduction proteins involved in the initiation of innate immune response, and by directly targeting mRNAs that encode specific inflammatory mediators, miRNAs can have an important impact on the magnitude of the ensuing inflammatory response.

The regulation of inflammation by miRNAs is primarily through altered expression of specific miRNAs in stimulated immune- or bystander cells (48). There is also evidence that the biogenesis of miRNAs is regulated as part of the inflammatory response, by altering the transcription, processing or stabilization of mature or precursor miRNA transcripts (40, 49). The initiation, spread, and resolution steps of inflammation are subject to both positive and negative regulatory events *via* miRNAs (50). The positive feedback initiates a cascade of molecular events that serve to combat against invasion of microbial pathogens and successful repair of tissue damage. The negative feedback, which is activated only during severe inflammation, is vital for preventing potentially damaging end-stage processes and maintaining tissue homeostasis (Figure 3). miRNA exerts their anti-inflammatory functions *via* multiple pathways, which are discussed in details below (Figure 4).

### miR-10a

This miRNA and its actions are well conserved among vertebrates and found to be an important posttranscriptional mediator in the control of inflammation (51). Importantly, its downregulation has been reported in inflammatory disorders such as rheumatoid arthritis (RA), inflammatory bowel disease (IBD), colitis, acute pancreatitis, and atherosclerosis (52–56).

In RA patients, miR-10a is downregulated by tumor necrosis factor alpha (TNF-α) and interleukin (IL)-1β, through promoting the production of the transcription factor YY1, a downstream gene of nuclear factor-κB (NF-κB). The downregulated miR-10a accelerates inhibitor κB (IκB) degradation and NF-κB activation. This is *via* targeting interleukin-1 receptor-associated kinase 4, transforming growth factor beta (TGF-β)-activated kinase 1 (TAK1), the beta-transducin repeat containing E3 ubiquitin ligase (β-TrCP), and mitogen-activated protein 3 kinase 7 (MAP3K7) that are key regulators of NF-κB signal transduction (56). In IBD patients, miR-10a regulates the pathogenesis by inhibiting DCs expression of IL-12/IL-23p40 and NOD2, as well as by inhibiting Th1 and Th17 cell function, thereby its aberrant expression plays a role in the progression of IBD (55). This miRNA is predominantly expressed in the intestines and contributes to the maintenance of intestinal homeostasis as described earlier. Mice with colitis express higher levels of IL-12/IL-23p40 and lower levels of intestinal miR-10a compared with control mice. In the era of much focus on the gut microbiome and its relation to disease development, it is interesting that an unbalanced intestinal microbiota may negatively regulate DCs miR-10a expression *via* TLR–TLR ligand interactions through the MyD88-dependent pathway (53). In acute pancreatitis, the decreased serum level of miR-10a may be related to the changes of immune homeostasis during disease progression (54). Furthermore, the differential expression of miR-10a contributes to the regulation of pro-inflammatory endothelial phenotypes in regions susceptible for atherosclerosis *in vitro* and *in vivo* by targeting MAP3K7 and β-TrCP (52). miR-10a is also expressed in Treg cells, indicating a role of this miRNA in Treg stability and function (57).

Considering that miR-10a inhibits multiple target genes involved in NF-κB signaling and is important in the pathogenesis of inflammatory diseases, manipulation of this miRNA expression level may provide a clinically applicable therapy. As it is downregulated in inflammatory conditions, targeting inflammatory responses through miR-10 mimic could be effective. Furthermore, we speculate that the expression level of miR-10a potentially can be used as a prognostic indicator for uncontrolled inflammation, but this would need more research.

### miR-21

Recent studies have revealed an essential role for miR-21 in the resolution of inflammation by negative feedback of inflammatory pathways (58–60). miR-21 acts as a negative modulator of TLR4 signaling by targeting the programmed cell death 4 (PDCD4) (58). Overexpression of miR-21 in macrophages leads to reduced secretion of IL-6 and increased IL-10 production, implying an anti-inflammatory effect (59). Importantly, miR-21 has a role in establishing the fine balance between Th1 and Th2 responses; treatment of miR-21-deficient DCs with lipopolysaccharide (LPS) resulted in an enhanced production of IL-12. Similarly, stimulation of miR-21-deficient CD4^+^ T cells with ovalbumin increased interferon (IFN)-γ and decreased IL-4 production (61). miR-21 also negatively regulates LPS-induced lipid accumulation and inflammatory responses in macrophages by modulating the TLR4–NF-κB pathway, indicating its potential application as a therapeutic agent for prevention and treatment of atherosclerosis (59). In line with that, deficiency of miR-21 in macrophages promotes endothelial inflammation during atherogenesis (62). Likewise, overexpression of miR-21 suppresses the macrophage inflammatory M1 phenotype and enhances the anti-inflammatory M2 phenotype (63). Importantly, elevated miR-21 expression promotes resolving inflammation following macrophage-mediated injury by targeting the phosphatase and tensin homolog and PDCD4 genes, which results in an anti-inflammatory phenotype and elevated production of IL-10 (64). This miRNA could potentially serve as translational biomarkers for detection of kidney injury and could be involved in the inflammatory response in relation to the pathogenesis of renal disease and tissue repair process (65). In this regard, miR-21 inhibits TNF-α-induced CD40 expression in renal cells *via* the SIRT1–NF-κB signaling pathway (60) and inhibits autophagy by targeting Rab11a in an *in vivo* model (66). These findings highlight miR-21 as one of the factors that controls the magnitude of inflammation and adds to our understanding of the regulation of the inflammatory processes. This might ultimately lead to targeted therapy for inflammatory disorders, particularly in diseases where macrophages have a central role. Application of miR-21 mimics and applies novel delivery methods that can be helpful to target macrophages in inflammatory diseases. Successful delivery of miRNAs is still a challenging task. However, novel approaches have improved the potential to deliver oligonucleotides that mimic miRNA expression and provide small molecules to improve and upregulate miRNA function.

### miR-24

miR-24 belongs to the miR-23~27~24 cluster and decreases NF-κB nuclear translocation and DNA binding, and TNF-α and IL-6 production mainly through suppressing the high mobility group box 1 (HMGB1)/NF-κB-associated inflammatory signaling (67). In murine models and human aortic tissue, miR-24 acts as a key regulator of endothelial inflammation and limits aortic vascular inflammation in a chitinase 3-like 1 (Chi3l1)-dependent modulation (68). This miRNA also regulates cytokine production in macrophages through targeting Chi3l1 (68). According to Jingjing et al., miR-24 overexpression significantly decreases the production of M1 phenotype markers such as iNOS, IL-6, TNF-α, CD86, and CD80 but increases the production of M2 markers such as Arg1, CCL17, CCL22, CD163, and CD206 in stimulated macrophages (69). Moreover, miR-24 exerts anti-inflammatory action by inhibition the production of pro-inflammatory cytokines in LPS-stimulated macrophages (70), and secretion of inflammatory mediators including TNF-α, IL-6, and IL-12p40, in response to infection through modulation of various genes involved in pathogen recognition and downstream signaling (71). In a mice model of asthma, miR-24 expression restricts Th2 cell differentiation over a wide range of IL-4 doses, and in mice without miR-24, T cells show enhanced allergic airway hypersensitivity and inflammatory responses (72). These results suggest that overexpression of miR-24 by using miRNA mimics may, in the future, be of therapeutic benefit in vascular inflammation, and inflammatory disorders associated with macrophages, as well as allergic airway hypersensitive inflammation. However, altering the expression level of a single miRNA can lead to changes of hundreds of genes, suggesting careful consideration of unwanted side effects.

### miR-124

Recently, miR-124 was discovered as a negative regulator of inflammation by targeting several pathways such as the signal transducer and activator of transcription (STAT) and TLRs. Its downregulation has also been reported in RA patients (73). The expression of miR-124 is significantly reduced in intestinal macrophages in pediatric intestinal failure patients in contrast to overexpression when miR-124 inhibits intestinal inflammation through attenuating production of IL-6 and TNF-α *via* targeting STAT3, a major factor in inflammatory response, and acetylcholinesterase, a negatively regulator of the cholinergic anti-inflammatory signal (74). Sun et al. reported that miR-124 targets STAT3 to decrease IL-6 production and TNF-α converting enzyme believed to reduce TNF-α release in response to LPS (75). Importantly, children with active ulcerative colitis have reduced levels of miR-124 and elevated levels of STAT3 in their colon tissues, which promote inflammation and pathogenesis of the disease (76). The miR-124 expression is enhanced in the peripheral leukocytes of patients with pulmonary tuberculosis, and MyD88 overexpression and/or infection induce its expression *in vitro*. Conversely, miR-124 negatively regulates multiple components of the TLR signaling, including TLR6, MyD88, TNF-α, and TNF receptor-associated factor 6 (TRAF6) (77), indicating an underlying negative feedback loop between miR-124 and TLRs signaling to prevent excessive inflammation (78). A decrease in miR-124 expression also contributes to an epigenetically reprogrammed, highly proliferative, migratory, and inflammatory phenotype of hypertensive pulmonary adventitial fibroblasts in calves and humans (79).

Interestingly, miR-124 expression is enhanced during allergic inflammation, thereby contributing to the development and maintenance of anti-inflammatory M2 phenotype (80). Furthermore, this miRNA negatively regulates LPS-induced TNF-α production in mouse macrophages by targeting ubiquitin-specific protein (USP) 2 and USP14, which control protein stability (81). Moreover, miR-124 inhibits experimental autoimmune encephalomyelitis and reduces neuroinflammation through inactivation of macrophages, and myelin-specific T cells *via* the C/EBP-α–PU.1 pathway (82). Importantly, peroxisome proliferator-activated receptor gamma (PPARγ), a member of the nuclear receptor superfamily, exerts its anti-inflammatory effects by upregulation of miR-124 through binding to its promoter region. This is important for PPARγ-mediated inhibition of pro-inflammatory cytokines production such as TNF-α and IL-6 (83). miR-124 also seems to be involved in morphine inhibition of innate immunity by directly targeting NF-κB and TRAF6 (84).

These data suggest that miR-124 may be of diagnostic value for inflammatory disease detection and severity prediction. Further investigations are needed to confirm and elucidate miR-124 implication in human immune-associated diseases, and hopefully, in the future, it may be possible to develop new therapeutic methods for treatment of inflammatory disorders.

### miR-145

A recent report found that loss of miR-145-induced pro-inflammatory signals in the innate immune response and was downregulated in ulcerative colitis (85). This miRNA inhibits release of IL-6 and CXCL8 in airway smooth muscle cells in patients with chronic obstructive pulmonary disease by targeting the mothers against decapentaplegic homolog 3 (SMAD3), a key element of the TGF-β1 inflammatory pathway (86). Furthermore, miR-145 functions to modulate expression of SMAD3 changes in downstream target genes expression, and IL-1β-induced extracellular membrane degradation in chondrocytes from osteoarthritis patients (87). The toll/interleukin-1 receptor domain-containing adaptor protein and TRAF6 are also targets for miR-145 suggesting an anti-inflammatory action for this miRNA (88). Interestingly, miR-145 seems to be involved in the anti-inflammatory effects of aspirin in atherosclerosis disease as seen *in vitro* by inhibiting the expression of CD40 (89). Inhibition of CD40 suppresses inflammatory factor production that is triggered by hypoxia such as IL-1β, TNF-α, and IL-6 (90). Besides, pomegranate polyphenolics attenuate inflammation and ulceration in experimental intestinal colitis by suppressing the p70S6K1/HIF1α signaling pathway, which is mediated in part through upregulation of miR-145 (91).

### miR-146

The miR-146 family comprises two genes, miR-146a and miR-146b, which are expressed in response to pro-inflammatory stimuli as negative feedback to control excessive inflammation (92). Their aberrant expression is associated with various inflammatory disorders such as RA, lupus disease, psoriasis, and osteoarthritis (93). Pharmacological studies have shown that NF-κB plays a critical role in the induction of miR-146 transcription, and MEK-1/2 and JNK-1/2 act in posttranscriptional processing to mature miRNA (94). Both miR-146a and miR-146b can regulate the inflammatory process by directly targeting TLRs and their downstream effectors, IRAK1 and TRAF6 (95). Importantly, miR-146a negatively regulates the IFN response (96), and the adaptive immunity by targeting adaptor protein (AP)-1 activity and IL-2 expression (97), as well as immune cell activation and cytokines production (98). Furthermore, miR-146b regulates diabetes-related retinal inflammation by suppressing adenosine deaminase 2 (99).

A recent study revealed downregulation of miR-146a in renal tissues of lupus nephritis, which was associated with increased expression of TRAF6 and NF-κB. miR-146a inhibits NF-κB transcriptional activity, biosynthesis of IL-1β, IL-6, IL-8, and TNF-α, and alleviates chemotactic effects toward macrophages *via* inhibition of TRAF6 activity (100). Tang et al. found that low expression of miR-146a contributed to lupus pathogenesis by overactivation of the IFN pathway. This miRNA directly inhibits the transactivation downstream of IFN such as IFN regulatory factor 5 and STAT1 (101). In addition, miR-146a attenuates sepsis-induced cardiac dysfunction by preventing NF-κB activation, inflammatory cell infiltration, and cytokine production *via* targeting of IRAK and TRAF6 in both cardiomyocytes and macrophages (102).

miR-146a and miR-146b expression in IL-β-stimulated human alveolar epithelial cells attenuate the release of IL-8 and RANTES after their transcription and not through targeting IRAK1 and TRAF6, which implies their action upon chemokine translation (25). miR-146a and miR-146b expression also induced in endothelial cells upon exposure to pro-inflammatory cytokines that inhibit the endothelial inflammatory response by inhibition of pro-inflammatory transcription activation, including the NF-κB, AP-1, and MAPK/EGR pathways. In addition, they modulate posttranscriptional pro-inflammatory pathways in endothelial cells *via* targeting the RNA binding protein HuR, indicating another way to control inflammation (103). Importantly, miR-146a is highly expressed in Treg cells and may therefore be critical for the ability of Treg to restrain IFN-γ-mediated pathogenic Th1 inflammatory responses. In these cells, miR-146a mediates downregulation of STAT1, a key transcription factor required for Th1 effector cell differentiation, and necessary for Treg ability to suppress Th1 responses (104).

According to Echavarria et al., prolonged exposure to angiopoietin-1, a vascular growth factor, leads to upregulation of miR-146b that inhibits angiopoietin-1 through selective targeting of IRAK1 and TRAF6. Also, it inhibits a wide array of LPS-induced responses such as leukocyte adhesion molecule expression, pro-inflammatory cytokine production, p38 and SAPK/JNK phosphorylation, and NF-κB activation (105). Moreover, apolipoprotein that binds lipids to form lipoproteins like LPS, suppress NF-κB-mediated inflammation and atherosclerosis by increasing miR-146a in damaged monocytes and macrophages leading to irreversible arrest of proliferation, *via* enhancement of transcription factor PU.1 (106). Also, miR-146a modulates pro-inflammatory signaling negatively *via* inhibition IL-6 and VEGF-A expression, at least in pigment epithelial cells (107), and by IL-6 and IL-8 in human fibroblasts (108).

miR-146a expression is induced both in macrophages and in mice after mycobacterial infection, and further suppresses the iNOS expression and NO generation *via* NF-κB and MAPK signaling and TRAF6 (109). This upregulation of miR-146a induces negative feedback of NF-κB signaling through targeting IRAK1 and TRAF6 (110). Thereby, levels of pro-inflammatory cytokines TNF-α, IL-1β, IL-6, and chemokine MCP-1 are reduced with subsequently facilitated replication of microbes such as mycobacteria (111). Also, the upregulation of miR-146a induced by viruses in human microglial cells leads to suppression of NF-κB activity and disruption of antiviral JAK–STAT signaling, which besides the anti-inflammatory activity helps the virus to evade from the immune response (112). miR-146a upregulation in viral infection acts as a negative regulator for the RIG-I-dependent type I IFN production by targeting TRAF6, IRAK1, and IRAK2 (113).

The identified actions indicate that an enhanced miR-146a and miR-146b expression may have, could identify a future therapeutic possibility for treatment of inflammatory disorders, as well as a potential target for control of viral or bacterial infections through inhibition of immune suppressive effects. The application of nanotechnology paves a new path in the development of effective delivery involving miRNAs.

### miR-149

miR-149 is a novel immune modulator of the innate immune responses. Its overexpression in macrophages has been linked to a significant decrease in MyD88 protein expression, as well as a reduced production of inflammatory mediators such as NF-κB, TNF-α, and IL-6 in response to infection or LPS stimulation (114). In addition, miR-149 inhibits the hepatic inflammatory response through STAT3-mediated signaling pathway (115). TNF-α induces endothelial activation through downregulation of miR-149, and its mimic transfection counteracted the TNF-α-induced expression of MMP-9, iNOS, and IL-6 (116). Consistently, downregulation of miR-149 has been linked to osteoarthritis chondrocytes; a joint disease that is caused by uncontrolled inflammatory immune responses (117). These findings give relevant ideas for future treatment strategies and in the diagnosis of immune disorders related to TNF-α.

### miR-155

miR-155 exhibits both anti- and pro-inflammatory functions, depending on the simulant involved (118). Upregulation of this miRNA leads to attenuation of inflammatory pathways, and adjustment to lower inflammatory intensity (118). For example, the TNF-α-induced miR-155 serves as a negative feedback regulator in endothelial inflammation involved in atherosclerosis by targeting NF-κB P65 (119). Furthermore, overexpression of miR-155 reduces chronic inflammation and provides protection against atherosclerosis-associated foam cell formation by targeting calcium-regulated heat stable protein 1, which in turn diminishes the stability of TNF-α mRNA (120). miR-155 also inhibits inflammatory response *in vitro* by translational inhibition of MyD88 and the inositol 5′-phosphatase SHIP-1 in infected macrophages (121).

Various inflammatory mediators such as TNF-α and IL-6 are markedly increased in mice liver cells when miR-155 is lacking. Moreover, NF-κB signaling is activated when miR-155 is absent, *via* enhancing p65 and inhibitor-κB kinase ε expression (122). In mature DCs, miR-155 downregulates inflammatory cytokines production in response to microbial stimuli. This miRNA also targets the TLR/IL-1 inflammatory pathway and TGF-β-activated kinase-1-binding protein 2 (TAB2), an adaptor in the TLR/IL-1 signaling cascade (123). Activation of miR-155 during septic lung injury alleviates inflammation through inhibition of TAB2, which in turn triggers autophagy (124). In addition, miR-155 inhibits IL-13-induced expression of eosinophilic chemokines CCL11 and CCL26 in human bronchial epithelial cells (125). miR-155-deficient mice have reduced numbers of Treg cells, both in the thymus and periphery, possibly due to impaired development (126). These data demonstrate a broad function of miR-155 in inflammation and a potential utility as therapeutic target.

### miR-181 Family

Accumulating evidence indicates an essential role for the miR-181 family (miR-181a, miR-181b, miR-181c, and miR-181d) in endothelial inflammation *via* regulating critical signaling pathways, such as downstream NF-κB (127). This is relevant in endothelial cell activation and immune cell homeostasis. miR-181b targets importin-α3, a protein critical for NF-κB nuclear translocation in *in vitro* and *in vivo* models of vascular endothelium (128). In addition, miR-181 family negatively regulates TNF-α mRNA stability (129). This miRNA family seems to be important in neuroinflammation as observed in experimental models. Knockdown of miR-181 enhanced LPS-induced production of pro-inflammatory cytokines such as TNF-α, IL-6, IL-1β, and IL-8, while their overexpression resulted in a significant increase in the anti-inflammatory cytokine IL-10 (130). Importantly, miR-181a regulates inflammatory responses by directly targeting IL-1α and inhibition the production of inflammatory factors such as IL-1β, IL-6, and TNF-α in THP-1 cells (131). miR-181a also modulates IL-8, another important inflammatory cytokine of early immune responses (132). These results suggest that therapeutic targeting of the miR-181 family might be an effective way to control excessive inflammation, especially in vascular and neurological tissue.

### Other Anti-Inflammatory miRNAs

miR-9 expression increases in human monocytes and neutrophils upon NF-κB activation and acts as a feedback control of the NF-κB-dependent responses (133). This miRNA inhibits formation of the inflammasome and attenuates atherosclerosis-related inflammation, likely *via* targeting JAK1/STAT1 signaling (134). Furthermore, overexpression of miR-9 in the cerebral cortex around the infarcted area is associated with reduced NF–κB signaling pathway-related factors such as NF-κB p65, TNF-α, and IL-1β (135). Downregulation of miR-9 results in increased synthesis of pro-inflammatory mediators such as IL-1β, TNF-α, IL-6, and MCP-1 (136). Other miRNAs induced by TNF-α are miR-17-3p and miR-31 that target adhesion molecules E-selectin and ICAM-1, respectively (137). Lai et al. have recently shown that miR-92a negatively regulates TLR-triggered inflammation in macrophages by targeting MKK4 kinase, and that stimulation by TLR ligands decreases its expression (138). miR-99b targets TNF-α and TNF-α superfamily member 4 receptor genes and thereby regulates expression of various pro-inflammatory cytokines such as IL-6, IL-12, and IL-1β (139). In myelodysplastic syndromes both miR-99b and miR-125b levels correlate negatively with TLR2 and MyD88 expression (140). Overexpression of miR-126 significantly abrogates high glucose-induced secretion of pro-inflammatory cytokines such as IL-6, TNF-α, and CCL2 in human gingival fibroblasts, and promotes the production of IL-10 through targeting TRAF6 (141). Moreover, miR-126 suppresses inflammation and ROS production in human endothelial cells in a milieu of high glucose through modulating the HMGB1 expression (142).

Another miRNA, miR-132, potentiates cholinergic anti-inflammatory signaling by targeting acetylcholinesterase in LPS-treated alveolar macrophages (143). In the presence of acetylcholine, upregulation of miR-132 suppresses LPS-induced NF-κB nuclear translocation and production of STAT3 and phosphorylated STAT3, while its downregulation enhances NF-κB nuclear translocation (144). miR-142-3p regulates murine macrophages synthesis of pro-inflammatory NF-κB1, TNF-α, and IL-6, at least in part, through targeting IRAK1 gene and posttranscriptionally imposing an anti-inflammatory effect by downregulation IRAK1 protein expression (145). miR-187 expression is induced by the potent anti-inflammatory cytokine IL-10 in TLR4-stimulated monocytes that impacts on TNF-α mRNA stability and translation and decreases IL-6 and IL-12p40 expression *via* downregulation of IκBζ, a master regulator of the transcription of these latter two cytokines (146). In murine macrophages, miR-210 negatively regulates LPS-induced production of pro-inflammatory cytokines such as IL-6 and TNF-α by targeting NF-κB1 (147). miR-210 also imposes an anti-inflammatory effect in articular cavities in rats with osteoarthritis by targeting DR6 and inhibiting NF-κB signaling (148). Besides, TLR3 activation induces placental miR-210 *via* HIF-1a and NF-κBp50 leading to decreased STAT6 and IL-4 levels. This function may contribute to the development of preeclampsia (149). miR-223, the last miRNA presented here, modulates the inflammatory response in human gingival fibroblasts *via* targeting IKKα and MKP5 (150). It further suppresses TLR4 signaling in macrophages (151) and regulates intestinal inflammation *via* repression of inflammasome formation (152).

## Summary and Future Directions

Through the last years, we have experienced a growing interest in how miRNAs may act as modulators of inflammatory pathways and regulate host immune responses. Some miRNAs impact on important negative feedback loops, while others serve to amplify the response of the immune system by depressing inhibitors of the response. miRNAs target signal transduction proteins involved in the initiation of innate immune responses, and the variety of different miRNAs impact on the intensity of the inflammatory response. Identifying functionally relevant miRNAs associated with processes that attenuate inflammation, and their target networks should produce new knowledge that could provide new therapeutic strategies for inflammatory diseases.

MicroRNAs may be developed as potential targets for new therapeutic strategies in inflammatory diseases. Such miRNA-based therapies may be achieved through manipulation of endogenous miRNA levels by the delivery of miRNA inhibitors or mimic to change expression of target genes. Therapeutic modulation of miRNAs may have several advantages over alternative gene/protein targeting strategies, notably the ease with which these sequences can be synthesized. Moreover, one miRNA can have multiple target genes, which may be more beneficial than targeting multiple different genes individually. Alongside, the critical role of miRNAs in the regulation of inflammation and their potential to be targeted by new therapeutics, caution must be taken because excessive inhibition or overexpression of miRNAs might predispose patients to cellular abnormalities, impaired immunity, or even cancer.

Despite the advancement in miRNA-based therapies in clinical trials, there is still much to learn about how to transform these into effective, patient-compliant, and targeted drug delivery therapies. Importantly, it is risky to invest in miRNA therapeutics due to biological challenges, the cost of production and scale-up, and the anticipated clinical approval challenges. The main barrier to miRNA-based therapy is development of pharmaceutical strategies of low toxicity for targeted delivery to specific sites. In support of this, novel nanotechnologies and delivery methods are under development for efficient and effective delivery. While extracellular circulating miRNAs have shown a high level of stability in human blood and other body fluids, an ideal delivery method should protect the miRNAs from the circulatory nucleases and deliver mRNAs intact to the target site.

In summary, we have discussed a set of unique miRNAs with anti-inflammatory properties and their regulatory pathways (Table S1 in Supplementary Material). The expression levels of these miRNAs may offer promising diagnostic value and severity prediction of different inflammatory diseases since miRNAs are stable in human blood, detectable with high sensitivity/specificity methods and measurable *via* miRNA microarrays and qRT-PCR arrays. Their diagnostic value must be further investigated to elucidate the molecular mechanisms underlying miRNAs implication in inflammatory disease pathogenesis, and possibly to develop new therapeutic methods in the future. Further knowledge from *in vivo* animal models of inflammatory diseases and clinical studies will be valuable. Although miRNA-based therapy will have limitations, we anticipate that it will be considered in future strategies aimed at diagnosing and treating acute and chronic inflammatory disorders.

## Author Contributions

AT drafted and VS and BN revised the manuscript. AT and MT-R designed and depicted all figures. All the authors read and approved the final version of the manuscript.

## Conflict of Interest Statement

The authors declare that the research was conducted in the absence of any commercial or financial relationships that could be construed as a potential conflict of interest.

## Abbreviations

microRNAs, miRNAs, or miRs, short non-coding ribonucleic acid molecules; RISC, RNA inducing silencing complex; TLR, toll-like receptor; PRR, pattern-recognition receptors; DCs, dendritic cells; NK, natural killer; Th, T helper; Treg, regulatory T; RA, rheumatoid arthritis; IBD, inflammatory bowel disease; TNF-α, tumor necrosis factor alpha; IL, interleukin; NF-κB, nuclear factor-κB; IκB, inhibitor κB; IRAK4, interleukin-1 receptor-associated kinase 4; TGF-β, transforming growth factor beta; TAK1, TGF-β-activated kinase 1; β-TrCP, beta-transducin repeat containing E3 ubiquitin ligase; MAP3K7, mitogen-activated protein 3 kinase 7; PDCD4, programmed cell death 4; LPS, lipopolysaccharide; IFN, interferon; PTEN, phosphatase and tensin homolog; HMGB1, high mobility group box 1; Chi3l1, chitinase 3-like 1; STAT, signal transducer and activator of transcription; TACE, TNF-α converting enzyme; TRAF6, TNF receptor-associated factor 6; USP, ubiquitin-specific protein; C/EBP-α, CCAAT/enhancer-binding protein alpha; PPARγ, peroxisome proliferator-activated receptor gamma; SMAD3, mothers against decapentaplegic homolog 3; TIRAP, toll/interleukin-1 receptor domain-containing adaptor protein; AP, adaptor protein; ADA2, adenosine deaminase 2; IRF5, IFN regulatory factor 5; CARHSP1, calcium-regulated heat stable protein 1; IKKε, inhibitor-κB kinase ε; TAB2, TGF-β-activated kinase-1-binding protein 2; TNFRSF-4, TNF-α superfamily member 4.
