# Supplementary material for: Anti-Inflammatory MicroRNAs and Their Potential for Inflammatory Diseases Treatment
Source: Front Immunol. 2018 Jun 25;9:1377. doi: 10.3389/fimmu.2018.01377 (PMC6026627; doi:10.3389/fimmu.2018.01377)
Supplement: Supplementary file 1 [file table_1.DOCX]

**Table 1: Anti-inflammatory miRNAs and their effects**

| miRNAs | Effects | Ref |
| --- | --- | --- |
| miR-9 | Acts as a feedback control of the NF-κB-dependent responses by fine tuning the expression of NF-κB1 | Bazzoni et al., 2009 |
|  | Inhibits activation of the NLRP3 inflammasome and attenuates atherosclerosis-related inflammation through targeting JAK1/STAT1 signaling | Wang et al., 2017 |
|  | Decreases the expression of NF‑κB signaling pathway‑related factors including NF‑κB p65, TNF‑α, and IL‑1β | Liu et al., 2016 |
|  | Lack of its expression results in the upregulation of proinflamma­tory cytokines/chemokines such as IL‑1β, TNF‑α, IL‑6, and MCP‑1 | Yao et al., 2014 |
| miR-10a | Its downregulation promotes NF-κB activation and the production of downstream mediators such as IL-1β, IL-6, IL-8, MCP-1, and TNF-α | Mu et al., 2016 |
|  | Inhibits DC expression of IL-12/IL-23p40 and NOD2, and inhibits Th1 and Th17 cell function | Wu et al., 2015 |
|  | Contributes to the maintenance of intestinal homeostasis through inhibition of DC production of IL-12/IL-23p40. | Xue et al., 2011 |
|  | Its downregulation in acute pancreatitis relates to the changes of immune-homeostasis during disease progression | Liu et al., 2014 |
|  | In athero-susceptible regions its suppression contributes to endothelial proinflammatory NF-κB signaling by targeting MAP3K7 and β-TrCP | Fang et al., 2010 |
|  | Inhibition of its expression leads to reduced FoxP3 expression levels and its expression is lower in unstable ‘‘exFoxP3’’ T cells | Jeker et al., 2012 |
| miR-17 | TNF‑α induces its expression that regulates neutrophil binding to endothelial cells through targeting E-selectin | Suárez et al., 2010 |
| miR-21 | Negatively regulates TLR4 signaling through targeting of the PDCD4 | Sheedy et al., 2010 |
|  | Abrogates the secretion of IL-6 and increases IL-10 in macrophages | Feng et al., 2014 |
|  | Establishes a fine balance between Th1 and Th2 responses | Lu et al., 2011 |
|  | Negatively regulates LPS-induced lipid inflammatory responses in macrophages | Feng et al., 2014 |
|  | Macrophage deficiency of its expression promotes endothelial inflammation during atherogenesis | Canfrán‐Duque et al., 2017 |
|  | Suppresses macrophage inflammatory M1 phenotype and enhances anti-inflammatory M2 phenotype | Caescu et al., 2015 |
|  | Promotes resolving inflammation following injury by macrophages and targets PTEN and PDCD4 genes | Das et al., 2014 |
|  | Serve as translational biomarkers for detection of kidney injury and involved in the inflammatory response in relation to the pathogenesis of renal disease and tissue repair process | Saikumar et al., 2012 |
|  | Inhibits TNF-α-induced CD40 expression in renal cells via the SIRT1-NF-κB signalling pathway | Lin et al., 2017 |
|  | Inhibits autophagy by targeting Rab11a in an *in vivo* model | Liu et al., 2015 |
| miR-24 | Decreases NF-κB nuclear translocation and DNA binding, TNF-α and IL-6 production through suppressing HMGB1/NF-κB signaling pathway | Yang et al., 2016 |
|  | Acts as a key regulator of vascular inflammation and limits aortic vascular inflammation in a Chi3l1-dependent fashion | Maegdefessel et al., 2014 |
|  | Decreases the production of M1 phenotype markers, and increases the production of M2 markers in stimulated macrophage | Jingjing et al., 2017 |
|  | Inhibits the level of proinflammatory cytokines by macrophage in response to LPS | Fordham et al., 2015 |
|  | Inhibits the secretion of inflammatory mediators including TNF-α, IL-6, and IL-12p40 in response to infection | Naqvi et al., 2015 |
|  | Its expression in T cells restricts Th2 cell differentiation over a wide range of IL-4 doses | Pua et al., 2016 |
| miR-31 | TNF‑α induces its expression that regulates neutrophil binding to endothelial cells through targeting ICAM-1 | Suárez et al., 2010 |
| miR-92 | Regulates TLR-triggered inflammation in macrophages by targeting MKK4 kinase | Lai et al., 2013 |
| miR-99b | Targets TNF-α and TNFRSF-4 receptor genes and regulates expression of various proinflammatory cytokines such as IL-6, IL-12, and IL-1β | Singh et al., 2013 |
|  | Its level is negatively correlates with TLR2 and MyD88 expression | Gañán-Gómez et al., 2014 |
| miR-124 | Inhibits intestinal inflammation by attenuating production of IL-6 and TNF-α in macrophages via targeting STAT3 and AChE | Xiao et al., 2016 |
|  | Modulates LPS-induced cytokine production by targeting STAT3 and TNF-α converting enzyme (TACE) | Sun et al., 2013 |
|  | Its downregulation in colon tissues of children with active ulcerative colitis increases the expression and activity of STAT3 | Koukos et al., 2013 |
|  | Negatively regulates multiple components of TLR signaling cascade including TLR6, MyD88, TRAF6, and TNF-α | Ma et al., 2014 |
|  | Its low expression contributes to an epigenetically reprogrammed, highly proliferative, migratory, and inflammatory phenotype of hypertensive pulmonary adventitial fibroblasts | Wang et al. 2014 |
|  | Increases in M2 macrophage and upregulates several M2 markers and downregulates the M1 markers | Veremeyko et al., 2013 |
|  | Negatively regulates LPS-induced TNF-α production by targeting USP2 and USP14 | Sun et al., 2016 |
|  | Inhibits autoimmune encephalomyelitis and reduces neuroinflammation by systemic deactivation of macrophages | Ponomarev et al., 2011 |
|  | Participates in the anti-inflammatory effects of PPARγ | Wang et al., 2017 |
|  | Associates in morphine inhibition of the innate immunity by directly targeting a subunit of NF-κB p65 and TRAF6 | Qiu et al., 2015 |
| miR-125 | Its level is negatively correlates with TLR2 and MyD88 expression | Gañán-Gómez et al., 2014 |
| miR-126 | Abrogates high glucose-induced secretion of proinflammatory cytokines such as IL-6, TNF-α, and CCL2, and promotes the production of IL-10 through targeting TRAF6 | Wu et al., 2017 |
|  | Suppress inflammation and ROS production in endothelial cells treated by high glucose through modulating the expression of HMGB1 | Tang et al. 2017 |
| miR-132 | Potentiates cholinergic anti-inflammatory signaling by targeting acetylcholinesterase | Shaked et al., 2009 |
|  | Suppresses LPS-induced nuclear translocation of NF-κB and production of STAT3 | Liu et al., 2015 |
| miR-142 | Negatively regulates the production of NF-κB1, TNF-α, and IL-6 in the macrophages through targeting IRAK1 gene and IRAK1 protein expression | Xu et al., 2013 |
| miR-145 | Loss of its expression induces proinflammatory signals in the innate immune response | Pekow et al., 2012 |
|  | Negatively regulates proinflammatory cytokines IL-6 and CXCL8 release from airway smooth muscle cells in COPD by targeting SMAD3 | O'leary et al. 2016 |
|  | Affects IL-1β-induced extracellular membrane degradation in osteoarthritis chondrocytes | Yang et al., 2014 |
|  | Targets TIRAP and TRAF6 in the inflammatory signaling | Starczynowski et al., 2010 |
|  | Involves in the anti-inflammatory effects of aspirin in atherosclerosis by inhibiting the expression of CD40 | Guo et al. 2016 |
|  | Suppresses inflammatory factor production triggered by hypoxia such as IL-1β, TNF-α, and IL-6 via targeting CD40 expression | Yuan et al., 2017 |
|  | Affects the anti-inflammatory activity of pomegranate polyphenolics | Kim et al., 2017 |
| miR-146 | Regulates the inflammatory process through directly targeting TLRs and associated downstream signalling proteins such as IRAK1 and TRAF6 | Bhaumik et al., 2008 |
|  | Negatively regulates the IFN response | Cameron et al., 2008 |
|  | Modulates adaptive immunity by targeting AP-1 activity and IL-2 expression | Curtale et al., 2010 |
|  | Regulates immune cells activation, and interferes with cytokines production | Jurkin et al., 2010 |
|  | Regulates diabetes related retinal inflammation by suppressing ADA2 | Fulzele et al., 2015 |
|  | Inhibits NF-κB transcriptional activity and inflammatory factor synthesis such as IL-1β, IL-6, IL-8, and TNF-α | Zheng et al., 2017 |
|  | Directly represses the transactivation downstream of type I IFN such as IRF5 and STAT1 | Tang et al., 2009 |
|  | Attenuates sepsis-induced cardiac dysfunction by preventing NF-κB activation, inflammatory cell infiltration, and inflammatory cytokine production via targeting of IRAK and TRAF6 | Gao et al., 2015 |
|  | Attenuates the release of the inflammatory chemokines IL-8 and RANTES at a step following their transcription and not through the targeting of IRAK1 and TRAF6 | Perry et al., 2008 |
|  | Inhibits the endothelial inflammatory response by dampening the activation of NF-κB, AP-1, and MAPK/EGR pathways | Cheng et al., 2013 |
|  | Critical for the ability of Treg cells to restrain IFN-γ-mediated pathogenic Th1 responses and associated inflammation | Lu et al., 2010 |
|  | Inhibits LPS-induced leucocyte adhesion, adhesion molecule expression, proinflammatory cytokine production, p38 and SAPK/JNK phosphorylation, and NF-κB activation | Echavarria et al., 2015 |
|  | Apolipoprotein expression suppresses NF-κB-mediated inflammation and atherosclerosis by enhancing miR-146a expression | Li et al., 2015 |
|  | Regulates proinflammatory signaling pathways in senescence via inhibition the expression IL-6 and VEGF-A in the pigment epithelial cells | Hao et al., 2016 |
|  | Negatively regulates the senescence‐associated inflammatory mediators such as IL‐6 and IL‐8 | Bhaumik et al., 2009 |
|  | Suppress the iNOS expression and NO generation via targeting NF-κB and MAPK signaling and TRAF6 | Li et al., 2016 |
|  | Establishes as a negative feedback loop in NF-κB signaling through targeting IRAK1 and TRAF6 | Liu et al., 2014 |
|  | Reduces the induction of proinflammatory cytokines TNF-α, IL-1β, IL-6 and chemokine MCP-1 in mycobacterial infection | Li et al., 2013 |
|  | Its upregulation during viral infection suppress the NF-κB activity and antiviral Jak-STAT signaling | Sharma et al., 2015 |
|  | Its upregulation during viral infection acts as a negative regulator for the RIG-I-dependent type I IFN production by targeting TRAF6, IRAK1, and IRAK2 | Hou et al., 2009 |
| miR-149 | Decreases the MyD88 protein expression, and production of inflammatory mediators NF-κB1, TNF-α, and IL-6 in response to infection or LPS stimulation in macrophages | Xu et al., 2014 |
|  | Inhibits hepatic inflammatory response through targeting STAT3-meidated signalling pathway | Zhang et al., 2017 |
|  | Its downregulation in osteoarthritis chondrocytes correlates with increased expression of proinflammatory cytokines such as TNF-α, IL-1β, and IL-6 | Santini et al., 2014 |
|  | Protects against endothelial dysfunction by negative regulation of MMP-9, iNOS and IL-6 | Palmieri et al., 2014 |
| miR-155 | Negatively regulates inflammatory pathways, and adjust the inflammatory response into a controllable intensity | Duan et al., 2016 |
|  | Serves as a negative feedback regulator in endothelial inflammation involved in atherosclerosis by targeting NF-κB P65 | Wu et al., 2014 |
|  | Relieves chronic inflammation by a negative feedback loop and plays a protective role during atherosclerosis-associated foam cell formation via targeting CARHSP1 | Li et al., 2016 |
|  | Inhibits inflammatory response by translational repression of MyD88 and the inositol 5´-phosphatase SHIP-1 in infected human macrophages | Bandyopadhyay et al., 2014 |
|  | Absence of its expression increases levels of various inflammatory mediators such as TNF-α and IL-6 in livers of mice | Yuan et al., 2016 |
|  | Downregulates inflammatory cytokine production in response to microbial stimuli via targeting TAB2 | Ceppi et al., 2009 |
|  | Alleviates inflammation in septic lung injury in mouse and cell models by inducing autophagy via inhibition of TAB2 | Liu et al., 2017 |
|  | Inhibits IL-13-induced expression of eosinophilic chemokines CCL11 and CCL26 in bronchial epithelial cells | Matsukura et al., 2016 |
|  | miR-155-deficient mice have reduced numbers of Treg cells, both in the thymus and periphery, due to impaired development | Kohlhaas et al., 2009 |
| miR-181 | miR-181b regulates NF-κB downstream signaling and vascular inflammation by direct targeting of importin-α3 | Sun et al., 2012 |
|  | miR-181 family negatively regulates TNF-α mRNA stability | Dan et al., 2015 |
|  | Its knockdown enhances LPS-induced proinflammatory cytokines, and its overexpression results in a significant increases in the expression of the anti-inflammatory cytokine IL-10 | Hutchison et al., 2013 |
|  | miR-181a regulates inflammatory responses by directly targeting of IL-1α and inhibition the production of inflammatory factors such as IL-1β, IL-6, and TNF-α in THP-1 cells | Xie et al., 2013 |
|  | Directly binds to the 3′ UTR of IL-8, and modulates its levels | Galicia et al., 2014 |
| miR-187 | Directly targets TNF-α mRNA stability and translation, and indirectly decreases IL-6 and IL-12p40 expression via downmodulation of IκBζ | Rossato et al., 2012 |
| miR-210 | Negatively regulates LPS-induced production of proinflammatory cytokines such as IL-6 and TNF-α by targeting NF-κB1 in murine macrophages | Qi et al., 2012 |
|  | Decreases inflammation in articular cavity in osteoarthritis rats by targeting DR6 and inhibiting NF-κB signaling pathway | Zhang et al., 2015 |
|  | Decreases STAT6 and IL-4 levels | Kopriva et al., 2013 |
| miR-223 | Controls the inflammatory response via targeting IKKα and MKP-5 in periodontal tissue | Matsui et al., 2016 |
|  | Inhibits inflammation by suppressing TLR-4 signaling in macrophages | Wang et al., 2015 |
|  | Regulates intestinal inflammation via repression of the NLRP3 inflammasome | Neudecker et al., 2017 |
